# Optimization of Setpoint Conditions for Enhanced Biofabrication of Silver Nanoparticles Using *Helichrysum crispum* Extracts

**DOI:** 10.3390/nano14231916

**Published:** 2024-11-28

**Authors:** Lebogang L. R. Mphahlele, Patrick T. Sekoai, Oluwatoyin Joseph Gbadeyan, Veshara Ramdas, Santosh Ramchuran, Viren Chunilall, Malusi Mkhize

**Affiliations:** 1 Biorefinery Industry Development Facility, Council for Scientific and Industrial Research, Durban 4041, South Africa; toyin2good@gmail.com (O.J.G.); vchunillal@csir.co.za (V.C.); 2 Discipline of Chemical Engineering, University of KwaZulu-Natal, Durban 4041, South Africa; mkhizen7@ukzn.ac.za; 3 School of Life Sciences, University of KwaZulu-Natal, Private Bag X01, Scottsville 3209, South Africa; 4 Bioprocessing Group, Council for Scientific and Industrial Research, Pretoria 0001, South Africa; vmalapermal@csir.co.za (V.R.); sramchuran@csir.co.za (S.R.); 5 School of Life Science, University of KwaZulu-Natal, Durban 4041, South Africa

**Keywords:** Box–Behnken Design, optimization, nanoparticles, biomass valorization

## Abstract

This study investigated the optimization of setpoint conditions used for the enhanced biofabrication of silver nanoparticles (H.C-AgNPs) using *Helichrysum crispum* extracts. A Box–Behnken Design (BBD) model was used to evaluate the effects of reaction time, temperature, an *H. crispum* extraction volume, and a 0.1 M AgNO_3_ solution volume. A second-order polynomial regression equation was developed with a high R² of 0.9629, indicating that the model explained 96.29% of the variability in the data. The statistical significance of the model was confirmed with an F-value of 25.92 and a *p*-value of less than 0.0001. The optimal biofabrication conditions were determined to be a reaction time of 60 min, a temperature of 50 °C, an *H. crispum* extract volume of 10 mL, and a silver nitrate volume of 90 mL, achieving a peak absorbance of 3.007 a.u. The optimized conditions were experimentally validated, resulting in an absorbance of 3.386 a.u., reflecting a 12.6% increase. UV-Vis spectroscopy showed a distinct surface plasmon resonance (SPR) peak at 433 nm. XRD analysis confirmed a crystalline face-centered cubic (FCC) structure with a primary diffraction peak at 2θ = 38.44° (111 plane). SEM and EDS results confirmed a uniform size and high purity, while FTIR spectra confirmed the involvement of phytochemicals in nanoparticle stabilization. TEM analysis revealed a uniform particle size distribution with a mean size of 19.46 nm and a dispersity of 0.16%, respectively. These results demonstrate the importance of statistical tools in optimizing the setpoint conditions used in the biofabrication of AgNPs, which have applications in various fields.

## 1. Introduction

Nanotechnology is one of the fastest-evolving fields in science and technology, experiencing rapid global advancements. The global nanotechnology market is projected to reach around $2.4 million by 2028, with a compound annual growth rate (CAGR) of 8.4% between 2022 and 2028. Ongoing innovations and the growing application of nanotechnology across diverse industries drive this expansion [1,2]. A similar trajectory is observed in South Africa as the nanotechnology sector is gaining momentum and is supported by initiatives such as the South African Nanotechnology Initiative (SANi). SANi plays a pivotal role in promoting research, development, and collaboration within the nanotechnology community, aiming to position South Africa as a leader in this cutting-edge field [3,4,5]. The country’s focus on nanotechnology is aligned with global trends and is seen as a critical tool in achieving the Sustainable Development Goals (SDGs), particularly in areas such as clean water and sanitation, clean energy, and good health and well-being [6,7,8].

Despite significant advancements in nanotechnology, the field still grapples with challenges related to the production of nanoparticles that exhibit consistent sizes and a diverse range of shapes [9]. Researchers have employed various techniques to address the challenges, including physical, chemical, biological, and hybrid methods. Each of these approaches offers unique advantages in tailoring the properties of nanoparticles to meet specific application needs. However, traditional physical and chemical fabrication methods often face significant constraints, such as high production costs, environmental pollution, and potential biological hazards [10]. These issues raise concerns regarding sustainability and safety, prompting a shift towards greener alternatives, particularly biological methods, which utilize natural resources and processes to produce nanoparticles with minimal environmental impact [11].

The biofabrication of nanomaterials using forestry-based extracts presents a promising and environmentally friendly approach to nanoparticle production. This method leverages the natural reducing and stabilizing properties of plant-derived compounds found in biomass, allowing for the swift creation of nanoparticles through redox reactions [12,13]. The upcycling of this biomass through biorefinery routes is well-suited for this scientific endeavor as South Africa produces ~500,000 tons of forestry waste, and 90% of this waste is landfilled resulting in environmental hazards. With the advent of strict environmental laws that aim to completely prohibit the landfilling of organic waste, the government and private sectors are now eager to adopt circular bioeconomy principles as a means of addressing these socioeconomic concerns [14].

Biofabrication of metal nanoparticles utilizing forestry-derived residues has been successfully demonstrated in the literature, which underscores the viability and effectiveness of this sustainable approach. Research has shown that various biomass sources, such as leaves, bark, and wood residues, contain phytochemicals that can facilitate the reduction of metal ions to form nanoparticles. For instance, Karami et al. [15] investigated the preparation of Fe_3_O_4_ nanoparticles using *Prosopis farcta* biomass extracts, thus alleviating the environmental concerns and remediation associated with this biomass. Similarly, Abdel-Megeed et al. [16] studied the synthesis of nanoparticles using *Lemna minor* (duckweed) extract to showcase its potential as a sustainable biogenic source for nanoparticle production. The resulting nanoparticles demonstrated promising properties, highlighting their capability to effectively control *T. castaneum* and *S. oryzae* populations. Additionally, they offer an eco-friendly alternative to synthetic insecticides, reducing environmental and health risks.

*Helichrysum crispum*, a species from the diverse genus of flowering plants known for their aromatic and medicinal properties, presents a valuable resource for biosynthesis applications [17,18]. These plants consist of a wide spectrum of phytochemicals that are key in nanoparticle synthesis, and these include flavonoids, phenolic acids, terpenoids, alkaloids, and essential oils, which are also applicable in medicine and cosmetics [19]. Recent research has shown the efficacy of *Helichrysum* biomass in the biosynthesis of metal nanoparticles, including silver, gold, and copper nanoparticles. Studies have shown that extracts from various *Helichrysum* species can reduce metal ions to their nanoscale form, with the resulting nanoparticles’ size, shape, and stability being influenced by the specific phytochemicals present [20]. This was confirmed by Aydin Acar et al. [21] who produced nanoparticles using *H. arenarium* originating from flowers. Interestingly, the nanoparticles exhibited strong inhibitory effects on the growth of well-known pathogenic bacteria, such as *Escherichia coli*, *Staphylococcus aureus*, and *Enterococcus faecalis*. Similarly, Taghavizadeh Yazdi et al. [22] produced nanoparticles using *Helichrysum graveolens* extracts, which served as both a stabilizing and reducing agent. The synthesized nanoparticles exhibited anticancer activity against the colon cancer cell line (C26), with the effectiveness being dependent on both the duration of exposure and the concentration of the nanoparticles. While the use of *Helichrysum* in nanoparticle synthesis presents numerous opportunities, there are knowledge gaps that need to be filled. For example, the variation in plant extracts prompts scientists to apply optimal conditions tailored for the recovery of the key phytochemicals, resulting in improved nanoparticle biofabrication yields.

Against this background, this study aims to optimize the biofabrication of silver nanoparticles using *H. crispum* extracts through the application of Response Surface Methodology (RSM), via the Box–Behnken Design (BBD). This was achieved by optimizing the operational setpoint conditions used in the extraction of the key phytochemicals. The biofabrication conditions studied in this work were the reaction time, extraction temperature, extraction volume, and silver nitrate volume, respectively. To the best of our knowledge, this is the first study that explores the use of empirical modelling and optimization tools such as BBD to enhance the biofabrication of nanoparticles using *Helichrysum crispum* as a sustainable feedstock. Such knowledge is critical for the field of nanotechnology as it will provide research practitioners with the optimal sets of conditions that contribute to enhanced nanoparticle biofabrication, therefore improving workflow/throughput by eliminating the tedious and time-consuming experiments, while reducing the research costs. The outcomes of this study will also assist in the development of sustainable and cost-efficient methodologies for green-based nanoparticle synthesis by identifying the key operational variables applicable to the enhancements of nanoparticle yields.

## 2. Materials and Methods

### 2.1. Collection of Botanical Specimens

*H. crispum* plants were purchased from a local traditional market in Durban, South Africa. The leaves were separated from the twigs, washed with distilled water, and dried under a fume hood for 72 h as previously described by Aiyegoro and Okoh [23]. After drying, the leaves were ground using an industrial blender and stored at 4 °C.

### 2.2. Leaf Extract Preparation

The *H. crispum* extract was prepared by adding 5 g of ground leaves to 300 mL of distilled water in a 500 mL glass beaker as per the literature [23]. The mixture was stirred at room temperature for 72 h, during which the solution gradually turned light brown. Thereafter, the solution was filtered using Whatman No. 1 filter paper. The filtered extract was then stored at 4 °C. The extraction was performed under sterile conditions.

### 2.3. Fabrication of Silver Nanoparticles (H.C-AgNPs)

Silver nanoparticles were synthesized using the modified procedure of Taghavizadeh Yazdi et al. [22]. Briefly, a 10 mL of *H. crispum* extract was mixed with 90 mL of 0.1 M aqueous AgNO_3_ solution which serves as the precursor to silver ions (Ag⁺) [24], at ambient conditions. The mixture was heated to 50 °C, and after 60 min, the solution turned dark brown, signifying the formation of H.C-AgNPs [22]. The reaction was conducted under dark conditions to prevent silver nitrate’s photoactivation. The resulting nanoparticles were centrifuged at 9000 rpm for 30 min and then resuspended in sterile distilled water to remove any unbound biological material. The purified silver nanoparticles were collected and air-dried at room temperature, then stored at 4 °C for subsequent analysis.

### 2.4. Optimization of H.C-AgNPs Synthesis Using the Box–Behnken Design

The optimization of the green-mediated fabrication of silver nanoparticles was conducted using BBD, a statistical tool used in optimizing the setpoint parameters (independent variables) of various processes, which in turn contributes to high-process outputs (dependent variables) based on the input variables. BBD uses a spherical design with excellent predictability within the optimization space. In this study, the BBD was chosen on the basis that it typically requires less design points compared to the axial points of CCD; hence the number of experimental runs are less in BBD than in CCD. This choice aligns with our minimal input variables as corroborated in the literature [25]. In this work, the setpoint parameters (independent variables) that were investigated on the silver nanoparticle yield (dependent variables) were reaction time (A), temperature (B), *H. crispum* extract volume (C), and a 0.1 M AgNO_3_ volume (D). Each parameter was evaluated at three distinct levels: low (−1), medium (0), and high (+1). The specific ranges and levels for each experimental factor are shown in Table 1.

The BBD was used to generate a total of 29 experimental runs based on the varying setpoint conditions applicable to the biofabrication of nanoparticles (Table 2). Each experiment was conducted in triplicates, and the dependent process variable (nanoparticle yield) was measured spectrophotometrically at the surface plasmon resonance. The relationship between the dependent and independent variables was modeled using the BBD and is described by the following second-order polynomial regression Equation (1).

(1) $$y=\beta_{0}+\sum_{i=1}^{k}\beta_{i}x_{i}+\sum_{i=1}^{k}B_{ii}x_{i}^{2}+\sum_{i=1}^{k-1}\sum_{J=2}^{k}B_{ij}x_{i}x_{j}+\varepsilon$$

where *y* is the predicted response; *β*_0_ is the regression coefficient; and *β_i_*, *β_ii_*, and *β_ij_* represent the linear, quadratic, and interaction coefficients, respectively. *x_i_* and *x_j_* denote the coded levels of independent variables. *ε* is the random error. The accuracy of the chosen model in navigating the optimization space was determined using the Analysis of Variance (ANOVA) via the Design Expert Software (Stat Ease, Inc, Minneapolis, MN, USA) [26,27,28].

### 2.5. Characterization of the Nanoparticles

#### 2.5.1. Phytochemical Analysis

The phytochemical screening of an *H. crispum* leaf extract was conducted using a multi-shot pyrolyzer, EGA/PY-3030 D, (Frontier Lab, Fukushima, Japan) attached to an ultra-alloy capillary column (30 m × 0.25 mm, 0.25 μm). Approximately 100 to 150 μg of the sample were pyrolyzed at 550 °C for 20 s, and the interface temperature to the analytical column was set at 350 °C.

#### 2.5.2. UV–Visible Spectroscopy

The primary approach of confirming the H.C-AgNPs was achieved using ultraviolet-visible (UV-Vis) spectroscopy, performed on a Genesys 150 spectrophotometer within the 200–800 nm spectral range. The as-prepared H.C-AgNPs (50 μg/L) were dispersed in an aqueous medium through sonication, and the UV-Vis spectra of the samples were immediately recorded post-sonication to analyze the nanoparticle formation and stability [29].

#### 2.5.3. XRD Analysis

The crystallographic characteristics of the H.C-AgNPs were investigated using X-ray diffraction (XRD) on a PANalytical X’Pert PRO X-ray Diffractometer, operated at 40 mA with Cu-K(α) radiation (1.54 Å) as the primary irradiating source. The Debye–Scherrer equation was applied to the obtained crystallographic peaks to calculate the mean particle size, providing insight into their crystalline structure and size distribution.

#### 2.5.4. Scanning Electron Microscopy (SEM-EDS) Analysis

The chemical composition and surface morphology of the synthesized nanoparticles H.C-AgNPs were meticulously examined using Energy-Dispersive X-ray Spectroscopy (EDS) and Scanning Electron Microscopy (SEM). These analyses were performed with the SEM-EDS Phenom Pharos Desktop System (Thermo Scientific, Waltham, MA, USA), providing detailed elemental composition and surface structure of the H.C-AgNPs.

#### 2.5.5. FTIR Spectroscopy

The H.C-AgNPs were subjected to Fourier Transform Infrared (FTIR) analysis to identify the biomolecules and functional groups involved in the reduction and stabilization processes. FTIR measurements were performed using a Perkin Elmer Frontier FTIR spectrometer within the range of 400–4000 cm⁻^1^, allowing for the detection of characteristic absorption bands related to the functional groups on the nanoparticles’ surface.

#### 2.5.6. Transmission Electron Microscopy (TEM) Analysis

H.C-AgNPs’ shape and size were analyzed using a Transmission Electron Microscope (JEOL 1400 TEM). For TEM analysis, samples were prepared by drop-casting a dispersion onto carbon-coated copper grids, which were then allowed to dry at room temperature.

## 3. Results and Discussion

### 3.1. Model Interpretation Based on Input Variables

The experimental data from Table 2, obtained using the BBD model, were used to develop a second-order polynomial regression i.e., Equation (2). This equation delineates the relationship between the nanoparticle yield (absorbance) and the studied input variables of reaction time, temperature, the volume of the *H. crispum* extract, and the volume of 0.1 M AgNO_3_.

Y = 3.01 − 0.0349A + 0.1624B + 0.3579C − 0.2138D − 0.1672AB − 0.3268AC − 0.0238AD + 0.2155BC − 0.0365BD − 0.2925CD − 0.2533A^2^ − 0.4773B^2^ − 0.4666C^2^ + 0.0577D^2^ (2)

where Y represents the nanoparticle yield (absorbance) while A, B, C, and D represent the linear coefficients of reaction time, temperature, *H. crispum* extract volume, and 0.1 M AgNO_3_, respectively. AB to CD represents the interactive coefficients, while A^2^ to D^2^ are the quadratic coefficients.

The statistical significance of the model was evaluated using the ANOVA as detailed in Table 3. A coefficient of determination (R^2^) of 0.9629 was obtained, signifying that 96.29% of the variability in the data can be explained by the model. The statistical significance of the model is further corroborated by the F and *p* values of 25.92 and <0.0001, respectively. This high R^2^ value indicates that the model is highly effective in predicting the synthesis of silver nanoparticles under the experimental conditions used in this study [30].

**Figure 1:**
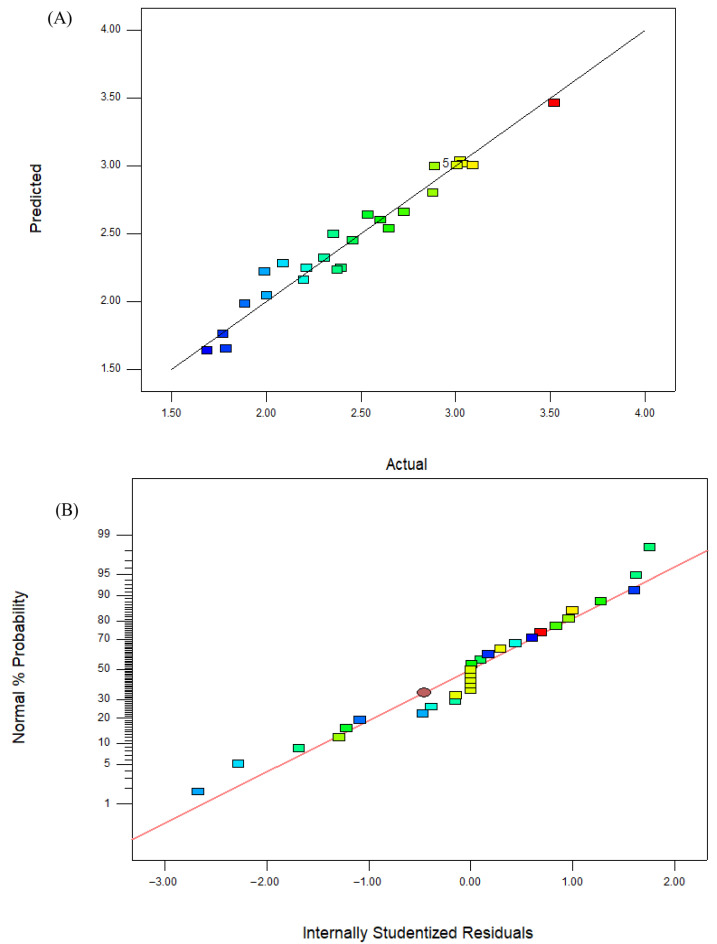
Depicting the schematic plot of the actual (experimental) versus predicted values (**A**) and the normal residual plot (**B**) of the H.C-AgNPs.

The adjusted R^2^ value of 0.9257, a coefficient of variation (C.V.%) of 5.21, a standard deviation of 0.13, a mean value of 2.54, and an adequate precision of 19.1950 collectively reinforce the validity of the results. These statistical metrics indicate a high level of reliability and consistency in the model’s predictions. Furthermore, Figure 1 demonstrates a strong correlation between the experimental yield and the yield predicted by the model, further affirming the model’s accuracy in capturing the behavior observed during the nanoparticle fabrication process.

### 3.2. Interactive Effect of Setpoint Conditions on Nanoparticle Synthesis

**Figure 2:**
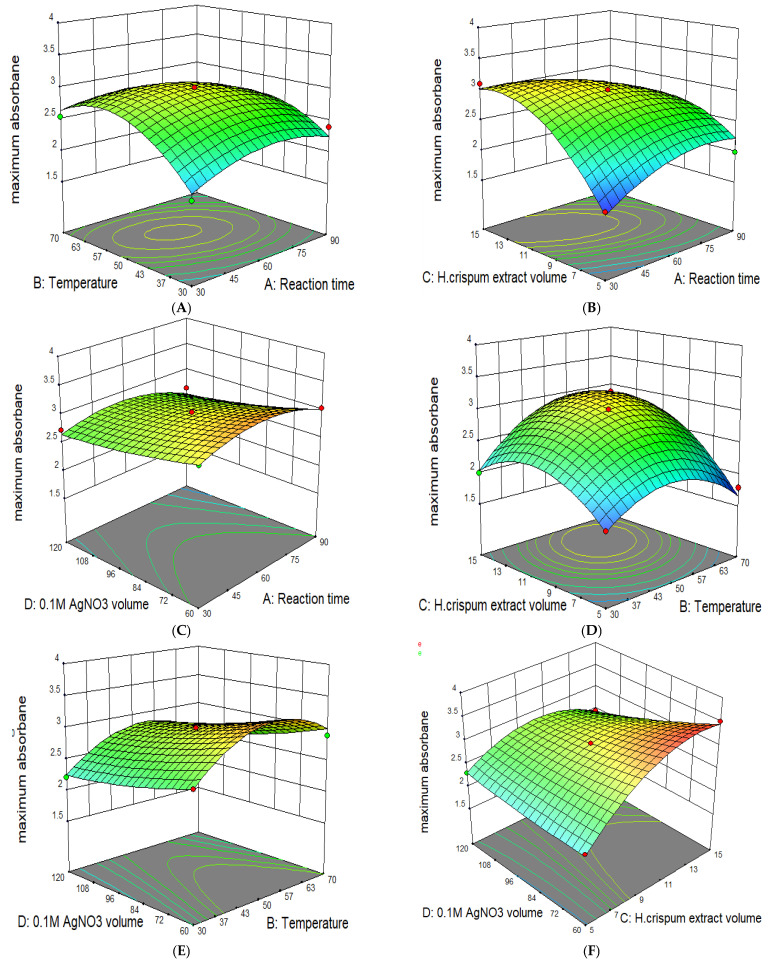
3D response surface plots showing the combined effects of parameters on the biofabrication of H.C-AgNPs.

**Figure 3:**
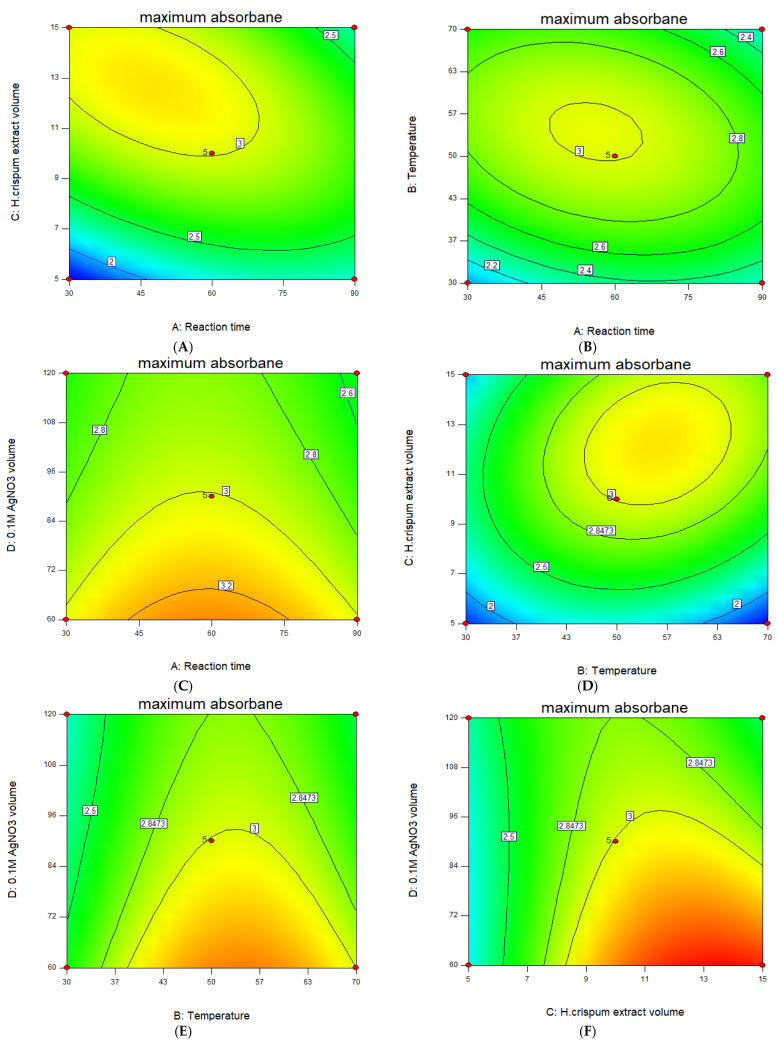
Contour plots showing the combined effects of parameters on the biofabrication of H.C-AgNPs.

The synergistic/pairwise effects of the studied variables on nanoparticle biofabrication response are represented by the 3D response surface plots (Figure 2) and their corresponding 2D contour map plots, which are the base region of the 3D response surface plots (Figure 3). These graphs show the behavior of the two variables within the experimental range on nanoparticle production response while the third variable remains constant.

#### 3.2.1. Effect of Reaction Time

The response surface graph illustrating the interactive effect of reaction time with other setpoint conditions on the yield of silver nanoparticles is shown in Figure 2A–C. Figure 2A demonstrates the combined influence of reaction time and temperature, with an increase in both variables leading to a peak absorbance of 3.007 a.u. at 60 min and 50 °C, indicating the optimal yield of silver nanoparticles is achieved within this search range. An increase in absorbance is likely due to the enhanced production of colloidal silver nanoparticles as reaction time increases [31,32]. Figure 2B shows a similar trend; however, the interaction with a 0.1 M AgNO_3_ volume (as also depicted in Figure 2C) had a lesser effect on absorbance. Additionally, the observed synergistic patterns were further revealed by the contour plots in Figure 3A–C, as these are the base regions of the 3D response surface curves for Figure 2A, B, and C, respectively.

#### 3.2.2. Effect of Temperature

The pairwise effect of temperature and other variables on the yield of nanoparticles is shown in Figure 2A,D,E. It was shown in these figures that lower temperatures (approximately 30 °C) contributed to a low absorbance which is likely attributed to the agglomeration of nanoparticles [32]. As the temperature increases, the absorbance also increases, indicating a higher yield of H.C-AgNPs. The response peaks resulting from the interaction of temperature with reaction time, the *H. crispum* extract volume, and the 0.1 M AgNO_3_ volume all showed maximum absorbance at 50 °C. However, shifting the temperature from 50 °C to 70 °C caused the maximum absorbance to decrease from 3.007 a.u. in Figure 2A to 2.301 a.u., and in Figure 2D, it decreased further to 1.743 a.u. Additionally, Figure 2E indicates a slight decline in absorbance with further temperature increases. This suggests the existence of an optimal temperature for nanoparticle synthesis beyond which thermal effects may hinder the process. Previous reports suggest that elevated temperatures can promote crystal growth around the nucleus, leading to decreased absorption [33]. These findings are in agreement with the literature as higher temperatures are reported to negatively impact nanoparticle yield [34]. Likewise, this pairwise behavior was further confirmed by the contour map plots in Figure 3A,D,E.

#### 3.2.3. Effect of *H. crispum* Extract Volume

The response surface plot illustrating the effect of the *H. crispum* extract volume is depicted in Figure 2B,D,F. The interaction between the extract volume with other setpoint variables, namely the reaction time, temperature, and a 0.1 M AgNO_3_ volume reveals that increasing the extract volume typically enhances the yield of silver nanoparticles. At the optimal volume of 10 mL, the absorbance peak indicates a higher yield of nanoparticles. However, beyond this volume, further increases lead to a decrease in absorbance, likely due to the saturation of active compounds or increased agglomeration. Figure 2B,D emphasize optimizing the extract volume in conjunction with reaction conditions to maximize nanoparticle yield. Figure 3B,D exhibit tightly packed circular contour lines, reinforcing the existence of an optimal point within the response surface. These circular contours illustrate a significant interaction between extract volume, reaction time, and temperature, with the highest response occurring at the center of the contours. Figure 2F illustrates the relationship between the extract volume and AgNO_3_ volume, revealing distinct trends in nanoparticle formation. As observed, increasing the extract volume results in higher absorbance values (3521 a.u) at 15 mL, indicating a more pronounced effect on nanoparticle formation and stabilization due to the elevated concentration of reducing and stabilizing agents in the extract [35]. This trend is also reflected in the contour plots (Figure 3F), where the curves display a steeper gradient of absorbance with rising extract volume, particularly at lower silver nitrate concentrations. This suggests that extract volume has a more significant impact on reaction efficiency and nanoparticle yield than silver nitrate volume alone.

#### 3.2.4. Effect of the 0.1 M AgNO_3_ Volume

The response surface diagram illustrating the effect of the 0.1 M AgNO_3_ volume is shown in Figure 2B,C,F. Increasing the AgNO_3_ volume from 60 to 120 mL did not result in significant changes in the absorption of the nanoparticles, although the optimal volume was found to be 90 mL. This indicates that the nanoparticle yield remained relatively stable even though higher AgNO_3_ volumes were tested, highlighting the importance of determining the optimal concentration for effective synthesis. The contour plots (Figure 3B,C,F) complement this finding and show relatively shallow gradients across the range of AgNO_3_ volumes. The contour lines remain close together, indicating the lack of significant interaction between the AgNO_3_ volume and other setpoint parameters. This highlights the importance of focusing on optimizing other conditions to improve the overall synthesis of silver nanoparticles.

Despite the positive results observed in the experiment, several challenges could be encountered during the optimization process. Variability in reaction time can lead to inconsistent nanoparticle formation due to differences in nucleation and growth rates, potentially altering the final yield. Temperature is another critical factor, as small fluctuations can influence the kinetics of the reduction process, affecting the size and stability of the nanoparticles. The volume of the *H. crispum* extract may introduce inconsistency in the concentration of active compounds, leading to variations in silver ion reduction efficiency. Similarly, variations in the 0.1 M AgNO_3_ volume can result in different concentrations of silver ions, which directly impacts the yield and size distribution of the nanoparticles. Ensuring precise control over these setpoint parameters is essential for improving the reproducibility and reliability of the results.

### 3.3. Optimization of Physicochemical Setpoint Conditions Using the BBD Model

To obtain the optimum physicochemical setpoint conditions that contribute to the enhanced nanoparticle biofabrication, the regression polynomial equation was solved using the method described by Myers and Montgomery [36]. The predicted BBD model shows that the maximum absorbance unit of 3.007 a.u. is achieved using a reaction time of 60 min, a temperature of 50 °C, an *H. crispum* extract volume of 10 mL, and a 0.1 M AgNO_3_ volume of 90 mL. Validation experiments were conducted using these sets of optimized conditions and produced an absorbance of 3.386 a.u., an improvement of 12.6%.

### 3.4. Characterization of Green Synthesized H.C-AgNPs

#### 3.4.1. Phytochemical Analysis

To obtain insights into the phytochemical constituents of *H. crispum* that contribute to the biofabrication of the H.C-AgNPs, pyrolysis-gas chromatography/mass spectrometry (Py-GCMS) was used to identify the key biomolecules. The preliminary phytochemical analysis (conducted in triplicates) of *H. crispum* leaf extract indicated the presence of various bioactive compounds such as phenols, flavonoids, etc. (Table 4). The tabulated compounds are known for their significant biological activities and contribute to the potential use of *H. crispum* extracts in green synthetic methods. The presence of these phytochemicals corresponds with the previous research findings of Zengin et al. [37].

#### 3.4.2. UV–Visible Spectroscopy

**Figure 4:**
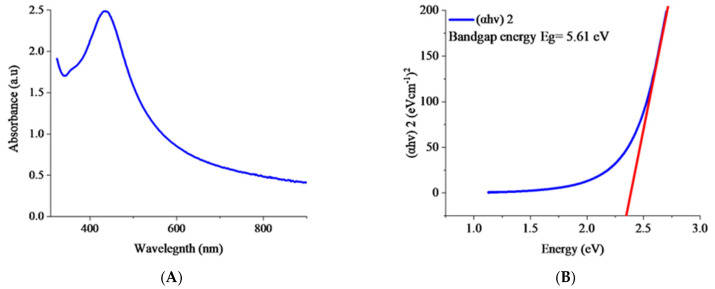
UV–visible spectrum (**A**) and Tauc plot (**B**) of H.C-AgNPs.

The biofabrication of H.C-AgNPs was monitored using UV-visible spectroscopy, as depicted in Figure 4A. The UV-Vis spectrum revealed a distinct absorbance peak at 433 nm, indicating the surface plasmon resonance (SPR) effect. This effect results from the collective oscillation of free electrons on the nanoparticle surface, which is influenced by the size of the nanoparticles and leads to specific absorption characteristics in the visible spectrum [38]. The peak at 433 nm corresponds to typical SPR peaks observed in similar studies [39] and is consistent with the near monodispersity of the nanoparticles, suggesting that the particles are predominantly uniform in size. Nevertheless, the slight broadening or shape distortion of the peak implies that there is some degree of aggregation present. Furthermore, the presence of a single SPR peak suggests that the nanoparticles are predominantly spherical [40]. The maximum wavelength was also used to calculate the energy gap (5.61 eV) which confirmed the optical properties of H.C-AgNPs.

(3) $${\left(ahv\right)}^{n}=A\left(hv-Eg\right)$$

where α is the absorption coefficient, *hν* is the photon energy (*h* is Planck’s constant, and *ν* is the frequency of light). *Eg* is the optical bandgap energy. *A* is a constant that relates to the properties of the material, and *n* is an exponent that depends on the type of electronic transition.

#### 3.4.3. XRD Analysis

**Figure 5:**
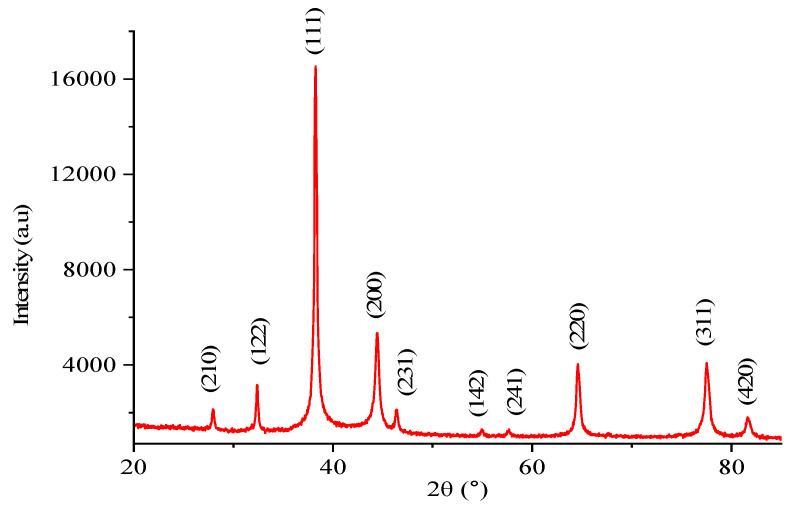
X-ray diffraction characterization of H.C-AgNPs.

The XRD analysis of H.C-AgNPs was largely consistent with the reference pattern for the face-centered cubic (FCC) crystal lattice system of silver nanomaterials, as reported in JCPDS Card No. 04-0783 [41,42]. The XRD pattern, shown in Figure 5, displays distinct diffraction peaks corresponding to the silver crystal planes. The Miller indices associated with these peaks confirm the crystalline nature of the AgNPs, with diffraction peaks observed at 2θ values of 27.64°, 32.34°, 38.44°, 44.34°, 46.32°, 54.96°, 57.71°, 64.59°, and 77.38°. These peaks correspond to the diffraction planes of (210), (122), (111), (200), (231), (142), (241), (220), and (311), respectively. The strong and sharp peak at 38.44°, which corresponds to the (111) plane, particularly confirms the well-defined crystalline structure of the AgNPs and their FCC geometry. Using the Debye–Scherrer’s equation (Equation (4)), an average particle size was calculated to be 20.51 nm., which is comparable to the TEM results shown in Figure 8.

(4) $$D=\frac{k\lambda }{\beta \cos\theta }\;(nm)$$

where *D* is the particle size, *k* = 0.9 is the shape factor, *λ* is the X-ray wavelength, *β* is the full width at half maximum (FWHM), and *θ* is the Bragg angle.

#### 3.4.4. Scanning Electron Microscopy (SEM-EDS) Analysis

**Figure 6:**
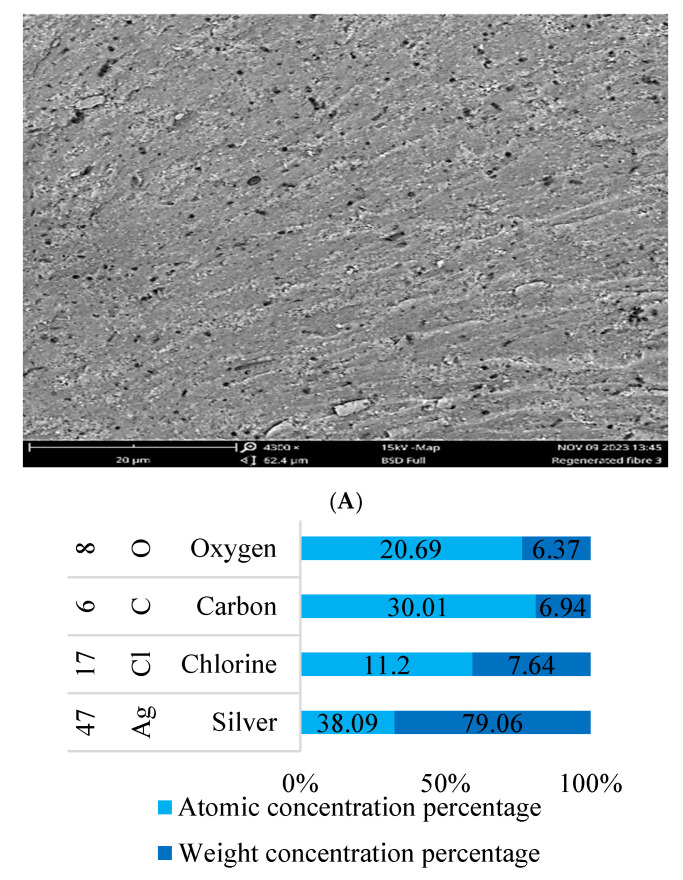

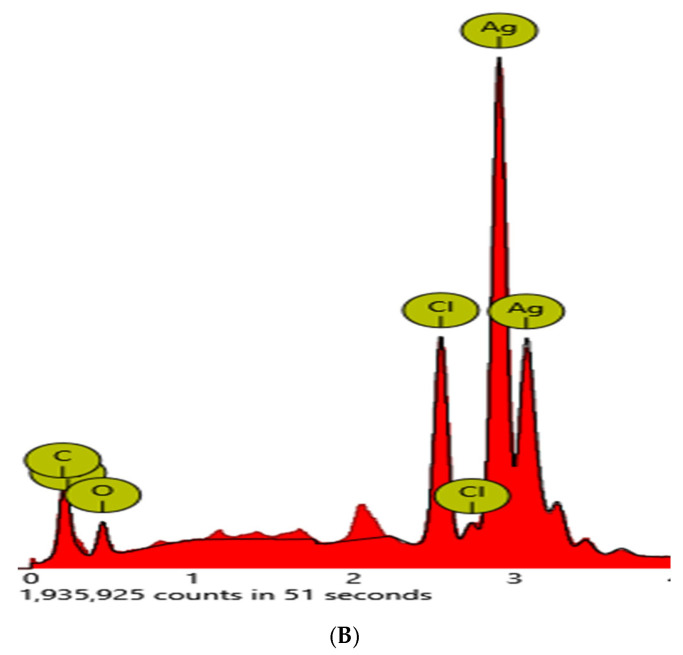
SEM image (**A**) and elemental analysis (**B**) of H.C-AgNPs.

The SEM image of H.C-AgNPs depicted in Figure 6A shows that the particles have a smooth surface with minimal roughness and tiny pores indicating uniform size [43]. The EDS analysis shown in Figure 6B identifies four primary elements: silver (Ag), carbon (C), oxygen (O), and chlorine (Cl). Silver is the most abundant element, with a weight content of 79.06% and an atomic content of 38.09%, confirming the successful synthesis of H.C-AgNPs. Carbon, present at 6.37% by weight and 20.69% by atomic percentage, likely comes from organic residues in the extract and the carbon tape used for mounting. Oxygen, at 6.94% by weight and 30.01% atomic percentage, may result from surface oxidation or adsorbed moisture. Chlorine, with a weight percentage of 7.64% and an atomic percentage of 11.20%, might originate from the *H. crispum* extract used during the synthesis process. This is likely due to the presence of specific phytochemicals containing chlorine in their molecular structures, such as 3-tert-Butyl-5-chloro-2-hydroxybenzophenone. These results suggest that the AgNPs are highly pure and have minimal contamination from other elements as similar EDS patterns were reported by Singh et al. [44].

#### 3.4.5. FT-IR Spectral Analysis

**Figure 7:**
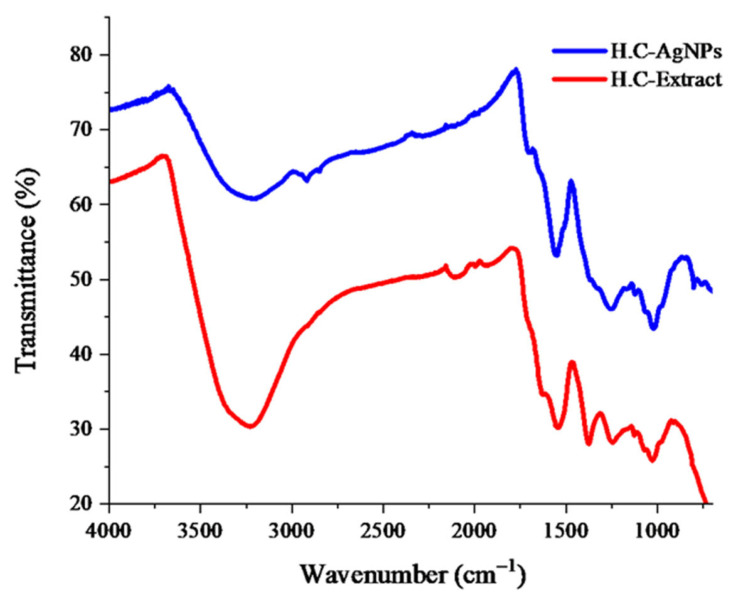
FTIR spectra of *H. crispum* extract and bioproduced H.C-AgNPs.

The FTIR spectra of the *H. crispum* extract and its corresponding silver nanoparticles (H.C-AgNPs) show remarkable changes in the vibrations of functional groups, indicating interactions between phytochemicals and silver nitrate (Figure 7). In the *H. crispum* extract, key peaks are observed at 3201 cm^−1^, 2098 cm^−1^, 1537 cm^−1^, 1384 cm^−1^, 1246 cm^−1^, and 1038 cm^−1^, corresponding to OH, NH, CH, CN (Amide II and III bands), and CO functional groups. In the FTIR spectrum of H.C-AgNPs, prominent peaks appear at 3259 cm^−1^, 2920 cm^−1^, 1562 cm^−1^, 1268 cm^−1^, 1012 cm^−1^, and 807 cm^−1^, showing slight shifts. The disappearance of the peak at 2098 cm^−1^ suggests the involvement of functional groups in the reduction and stabilization of AgNPs, while the new peak at 2920 cm^−1^ further supports this interaction. These spectral changes confirm the formation of silver nanoparticles through the reduction of silver ions by the bioactive compounds in the *H. crispum* extract [45]. This phenomenon is consistent with previous studies using plant extracts as reducing and stabilizing agents in the formation of silver nanoparticles, as reported by Masum et al. [46] and Balčiūnaitienė et al. [47].

#### 3.4.6. Transmission Electron Microscopy (TEM) Analysis

**Figure 8:**
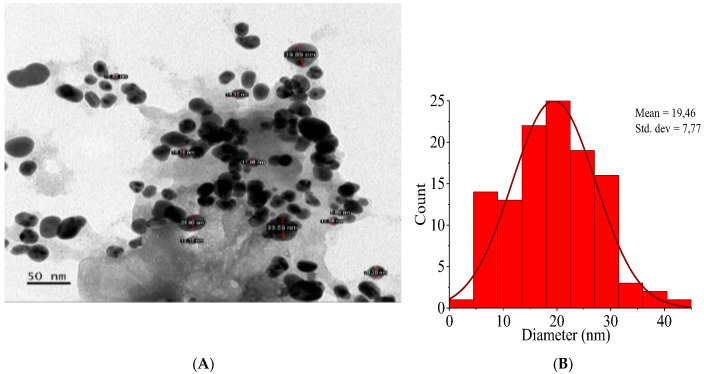
TEM image (**A**) and size distribution histogram (**B**) of H.C-AgNPs.

The morphological properties, particle size, and dispersity of H.C-AgNPs were investigated by TEM. As shown in Figure 8A, the TEM image showed that the nanoparticles predominantly have a spherical to hemispherical shape. The observed morphology is consistent with the expected shape for silver nanoparticles synthesized via phytochemical methods as reported in the literature [48,49]. The nanoparticle sizes range from 10.55 nm to 33.59 nm. Figure 8B illustrates a normal size distribution for the observed nanoparticles. The standard deviation of the particle size was 7.77 nm, with a mean particle size of 19.46 nm, which is closer to 20.51 nm according to the XRD results. This low standard deviation highlights the uniformity of nanoparticle sizes within the sample. Furthermore, the normal size distribution is supported by the nanoparticles’ dispersity, measured at 0.16%. The dispersity is a measure of the distribution width around the mean and is calculated as the ratio of the standard deviation to the mean particle size. A dispersity value of 0.16% indicates that the size distribution is quite narrow, which supports the observation of uniform particle sizes and confirms the high quality of the synthesized nanoparticles.

## 4. Conclusions

The study on the optimization of physicochemical setpoint conditions for silver nanoparticle biofabrication using *H. crispum* biomass extracts successfully demonstrated a viable approach to identifying the optimal sets of variables that contribute to improved nanoparticle yields. Application of the BBD model and subsequent polynomial regression provided a clear relationship between reaction variables and nanoparticle yield, with a high coefficient of determination (R^2^ = 0.9629) confirming the accuracy of the model. Statistical analyses, including ANOVA, highlighted the validity and reliability of the model. The optimal conditions identified through response surface diagrams revealed that a reaction time of 60 min, a temperature of 50 °C, an extract volume of 10 mL, and a silver nitrate volume of 1.0 mL were crucial for maximizing nanoparticle fabrication. UV-Vis spectroscopy, XRD, and TEM analyses further confirmed the synthesis process and revealed nanoparticles with a spherical morphology, a crystalline face-centered cubic structure, and an average size of 19.46 nm. Overall, these results highlight the potential of *H. crispum* extracts in producing high-quality silver nanoparticles under optimized conditions and offer significant prospects for applications in textile, water purification, wound healing, medical devices, and catheters to prevent and treat bacterial infections. Further studies could involve testing the nanoparticles in diverse environmental conditions and evaluating their long-term effectiveness and safety. Furthermore, scaling-up production and addressing any potential toxicity concerns should be prioritized to ensure broader applications in various industries. By expanding these areas of investigation, we can unlock the full potential of silver nanoparticles for commercial and scientific advancements. In addition, this study shows how the tenets of a circular bioeconomy could be leveraged by upcycling the overabundant plant-based extracts via innovative biorefinery routes for green-based nanoparticle synthesis.

## Figures and Tables

**Table 1:** Input variables used in the BBD model.

Variables	Units	Factors	Range and Levels		
			Low (−1)	Medium (0)	High (+1)
Reaction time	min	A	30	60	90
Temperature	°C	B	30	50	70
*H. crispum* extract volume	mL	C	5	10	15
0.1 M AgNO_3_	mL	D	60	90	120

**Table 2:** Experimental runs used in the biofabrication of H.C-AgNPs.

Run	A: Reaction Time (min)	B: Temperature (°C)	C: *H. crispum* Extract Volume (mL)	D: 0.1 M AgNO_3_ Volume (mL)	Absorbance (a.u)
1	60.00	50.00	5.00	120.00	2.306
2	30.00	50.00	5.00	90.00	1.689
3	90.00	30.00	10.00	90.00	2.396
4	60.00	30.00	5.00	90.00	1.773
5	60.00	70.00	10.00	60.00	2.890
6	30.00	70.00	10.00	90.00	2.537
7	90.00	50.00	15.00	90.00	2.089
8	60.00	30.00	10.00	120.00	2.215
9	30.00	50.00	15.00	90.00	3.092
10	60.00	50.00	10.00	90.00	3.007
11	60.00	70.00	15.00	90.00	2.881
12	60.00	50.00	5.00	60.00	2.199
13	60.00	50.00	10.00	90.00	3.007
14	30.00	50.00	10.00	120.00	2.728
15	90.00	50.00	10.00	120.00	2.648
16	60.00	70.00	10.00	120.00	2.356
17	90.00	70.00	10.00	90.00	2.375
18	90.00	50.00	5.00	90.00	1.993
19	60.00	50.00	15.00	120.00	2.458
20	30.00	50.00	10.00	60.00	3.024
21	60.00	50.00	10.00	90.00	3.007
22	90.00	50.00	10.00	60.00	3.039
23	60.00	30.00	10.00	60.00	2.603
24	60.00	70.00	5.00	90.00	1.789
25	60.00	50.00	10.00	90.00	3.007
26	60.00	50.00	15.00	60.00	3.521
27	60.00	50.00	10.00	90.00	3.007
28	30.00	30.00	10.00	90.00	1.889
29	60.00	30.00	15.00	90.00	2.003

**Table 3:** ANOVA of the derived BBD model.

Sources	Sum of Squares	df	Mean Squares	F-Value	*p*-Value	R^2^-Squared
Model	6.34	14	0.453	25.92	<0.0001	significant
A-Reaction time	0.0146	1	0.0146	0.8372	0.3757	
B-Temperature	0.3166	1	0.3166	18.11		
C-*H. Crispum* extract volume	1.54	1	1.54	87.97	<0.0001	
D-0.1 M AgNO_3_ volume	0.5483	1	0.5483	31.37	<0.0001	
AB	0.1119	1	0.1119	6.4	0.024	
AC	0.4271	1	0.4271	24.44	0.0002	
AD	0.0023	1	0.0023	0.1291	0.7247	
BC	0.1858	1	0.1858	10.63	0.0057	
BD	0.0053	1	0.0053	0.305	0.5895	
CD	0.3422	1	0.3422	19.58	0.0006	
A^2^	0.4163	1	0.4163	23.82	0.0002	
B^2^	1.48	1	1.48	84.57	<0.0001	
C^2^	1.41	1	1.41	80.81	<0.0001	
D^2^	0.0216	1	0.0216	1.23	0.2853	

df: degrees of freedom, F-value: Fisher–Snedecor distribution value, *p*-value: probability value, R^2^-squared: coefficient of determination.

**Table 4:** Components of *H. crispum* based on the preliminary aqueous leaf extract screening.

Ketone	Retention Time	Area%	Name
	2272	10.82	2-Propanone, 1-Hydroxy
	2477	3.53	2-Pentanone
	2.56	8.1	3-Pentanone
	2696	2.31	2-Butanone, 3-Hydroxy-
	3248	3.91	2-Pentanone, 3-Methyl-
	3898	1.08	Cyclopentanone
	4675	3.65	2-Cyclopenten-1-One
	6435	1.74	2-Cyclopenten-1-One, 2-Methyl-
	6954	1.14	3-Tert-Butyl-5-Chloro-2-Hydroxybenzophenone
	7659	0.88	2(5h)-Furanone, 5,5-Dimethyl-
	8089	1.62	2-Cyclopenten-1-One, 3-Methyl-
	9897	1.25	1,2-Cyclopentanedione, 3-Methyl-
Total		40.03	
Ester	Retention Time	Area%	Name
	2.05	2.8	Propanoic Acid, Methyl Ester
	5441	0.79	Acetic Acid Ethenyl Ester
Total		3.59	
Aldehyde	Retention Time	Area%	Name
	2204	1.52	2-Butenal
	3669	2.04	Butanedial
Total		3.56	
Alkene	Retention Time	Area%	Name
	2145	2.08	Hexatriene Isomer
	3501	5.39	Benzene, Methyl-
Total		7.47	
Amide	Retention Time	Area%	Name
	5.99	3.52	Acetamide
Total		3.52	
Phenols	Retention Time	Area%	Name
	8628	13.55	Phenol
	22,313	3.22	O-Cresol
	27,392	2.99	Phenol, 4-Methoxy-
	17,387	12.51	2,5-Cyclohexadiene-1,4-Dione, Compd. With 1,4-Benzenediol (1:1)
Total		32.27	
Alcohol	Retention Time	Area%	Name
	6555	0.91	Cyclohexane methanol Alpha. -(2-Methyl-2-Propenyl)-

## Data Availability

The raw data supporting the conclusions of this article will be made available by the authors on request.
